# Emergency calls and need for emergency care in patients looked after by a palliative care team: Retrospective interview study with bereaved relatives

**DOI:** 10.1186/1472-684X-7-11

**Published:** 2008-08-12

**Authors:** Christoph HR Wiese, Andrea Vossen-Wellmann, Hannah C Morgenthal, Aron F Popov, Bernhard M Graf, Gerd G Hanekop

**Affiliations:** 1 Department of Anaesthesiology, Emergency and Intensive Care Medicine, Medical Centre University of Goettingen, Goettingen, Germany; 2 Department of Palliative Medicine, Medical Centre University of Goettingen, Goettingen, Germany; 3 Department of Thoracic and Cardiovascular Surgery, Medical Centre University of Goettingen, Goettingen, Germany

## Abstract

**Background:**

During the last stage of life, palliative care patients often experience episodes of respiratory distress, bleeding, pain or seizures. In such situations, caregivers may call emergency medical services leading to unwanted hospital admissions. The study aims to show the influence of our palliative care team to reducing emergency calls by cancer patients or their relatives during the last six month of life.

**Methods:**

Fifty relatives of deceased patients who had been attended by our palliative care team were randomly selected. Data was obtained retrospectively during a structured interview. In addition to demographic data, the number of emergency calls made during the final six months of the patient's life, the reason for the call and the mental compound score (MCS-12) of the caregivers was registered.

**Results:**

Forty-six relatives agreed to the interview. Emergency calls were placed for 18 patients (39%) during the final six months of their lives. There were a total of 23 emergency calls. In 16 cases (70%) the patient was admitted to the hospital. Twenty-one (91%) of the calls were made before patients had been enrolled to receive palliative care from the team, and two (9%) were made afterwards. The mean mental compound score of the caregivers at the time of the interview was 41 (range 28–57). There was a lack of correlation between MCS-12 and number of emergency calls.

**Conclusion:**

Emergency calls were more likely to occur if the patients were not being attended by our palliative care team. Because of the lack of correlation between MCS-12 and the number of emergency calls, the MCS-12 cannot indicate that acutely stressful situations triggered the calls. However, we conclude that special palliative care programs can reduce psychosocial strain in family caregivers. Therefore, the number of emergency calls may be reduced and this fact allows more palliative patients to die at home.

## Background

The onset of acute symptoms during the final stage of a palliative patient's life can place an overwhelming psychosocial burden on the caregiving relatives. These facts can cause the caregivers to call for emergency medical services, which can result in patients being admitted to the hospital [1,2]. In order to avoid such situations, a framework of support by a palliative care team must be established early on, that gives patients and their relatives' access to competent advice and assistance at 24 hours a day. Although there is no definitive evidence for the effectiveness of such a support network, its importance has been repeatedly emphasized [3,4]. Emergency care oriented to the needs of the patient and the relatives could be considerably improved by a close cooperation between a palliative care team and the emergency physicians. For example, possibilities would be to integrate palliative care teams into the prehospital emergency care, palliative care availability twenty-four hours per day, and advanced care planning [5-7].

The type of prehospital care and particularly its appropriateness for the palliative patient still depends to a great extent on the training of the emergency physician [8]. An improvement in the emergency care of palliative patients could be achieved by amending the training curriculum for emergency medicine specialists [e.g. [9]]. Improved training of the primary care givers (physicians and nursing staff) as well as a closer cooperation between emergency and palliative medicine can optimize the patient-oriented care and reduce the number of unwanted hospital admissions [10,11]. This would make it more often possible to fulfill the patient's wish to "die at home" [12,13].

The present study aims to show the influence of our palliative care team during the last six months of life with regard to reducing the number of emergency calls by palliative cancer patients and their relatives. Therefore, the study approaches the important issue of whether and how our palliative care team may prevent unnecessary hospital admissions towards the end of life. Another aim of the study was to determine factors correlating with a tendency to call the emergency services. In addition, we investigated the relationship between psychosocial factors and the occurrence of overwhelming psychological strain in the caregiving family members. The emergency calls for the patients in the study are evaluated with regard to the type of care available at the time each call was placed and the type of medical intervention resulting from the call.

## Methods

Fifty caregiving relatives (overall number of patients died during the two-year time period was 250) were chosen to participate in this survey that was conducted with institutional review board approval (Note: equivalent to ethics approval in the UK). There were caregiving blood relatives included and other caregiving persons who function like family were also recruited. In the following all caregiving persons were declared as relatives. The relatives were randomly selected by a person not involved in the study. This person picked every fifth from the records of all patients who had been in palliative care at some time during a two-year period and who had since died. The death of the patient occurred at least two but less than 24 months before the beginning of the investigation. Concerning their cognitive status all relatives were capable of participating. All participants were required to be German speaking. To be included into the study the patient had to have been in the care of our palliative care team for at least two weeks and the relatives had to give their written consent. There were no exclusion criteria other than non-fulfillment of the inclusion criteria.

The randomly selected caregiving relatives were approached for the study by telephone. The interviews were scheduled after the relatives had given their oral consent. During a time period of two weeks after oral consent the relatives were interviewed by the investigator. Written consent was obtained prior to the interviews at which time the structure of the questionnaire was explained.

Data was obtained retrospectively by an interview using a questionnaire with structured questions. This questionnaire was specially designed for this study. Concerning demographics, cancer disease of the patient, relationship of the care-giving relatives with the patient, the chosen sample was representative of all patients and relatives who had been treated by our palliative care team in a home-care setting during the defined time period.

One investigator conducted the interviews. The investigator was no member of our palliative care team. The answers were recorded by the interviewer on an information sheet. The structured interview was conducted according to a standardised scheme (specific answers to very specific questions). Therefore, the answers of the interviewed relatives were limited. Further questions, comments and explanations were in the form of an open interview. The structured interview was scheduled to last 90 minutes. There was no time limit concerning the open questions after the structured interview was finished. The whole interview was conducted at caregiving relatives home.

The following data like reasons for agreeing to or for declining to participate in the interview, duration of the interview, average time to collecting the data, and demographics like age and gender of the patient and the caregiving relatives, the relationship of the caregiving person to the patient, cancer diagnosis, time when diagnosis of cancer was first confirmed, place of death, and duration of care by the palliative care team were recorded.

The following special aspects of the interview like number of emergency calls made during the final six month, reasons for the emergency calls, type of care at the time of the emergency situation, point of care when the emergency situation occurred, and subjective feeling of psychosocial strain experienced by the relatives while caring for the patient were recorded. The objective psychological strain on the caregiving relatives was of particular interest, notwithstanding the fact that this is difficult to determine accurately after intervals of differing lengths had passed since the event (emergency call). For this we obtained the subjective personal assessment of the interviewees as well as the retrospectively determined MCS-12 as an objective quantifier of psychological stressors.

The SF-12^® ^and its different scales (e.g. MCS-12, PCS-12) is a short instrument with good test characteristics and substantial validity. The SF-12^® ^estimates scores for four health concepts (physical functioning, role-physical, role-emotional, and mental health). The health survey is constructed using a Likert method of summated ratings. Furthermore we used a self constructed questionnaire to evaluate the relatives "Quality of Life" after the death of the patient. This questionnaire was not validated but we were able to evaluate the subjective psychosocial feeling of the caregiving relatives.

Emergency medical treatment was defined as a call placed to the local emergency service dispatcher requesting emergency assistance. Other calls were not included in the study.

All data relating to the patients and their family members were anonymised to protect their identity and prevent retrospective matching of persons and information. Ethical and data protection aspects were implemented according to the declaration of Helsinki [14,15]. Data were recorded and analyzed with MS Excel 2003 (Microsoft Inc.) and the statistics package SPSS 12.0 (SPSS Inc.). The Wilcoxon-Signed-Rank Test and Fisher's Exact Test were used to determine distribution and stochastic independence of the data as well as to test for significant differences. The significance level was designated as p < 0.05.

The questionnaire was evaluated with regard to its practicability in five test interviews that were not included in the study. As a result of this process, there were no revisions of the questionnaire necessary.

## Results

Forty-six relatives (92% of the initially selected sample) participated in the study. Four (8%) withheld their consent. The presented data refer only to the participating relatives. The demographic data of the patients and the caregiving relatives are shown in Table 1 and Table 2. The structured interview took an average time of 85 minutes (range 72–94 minutes). The whole interview took an average time of 124 minutes (range 98–285 minutes).

**Table 1:** Demographic data (Caregiving relatives)

	Caregiver
Sex	Number (%)
female	32 (70%)
male	14 (30%)

Age (years)	Mean (min/max)
	58 (22–80)

Caregiving person	Number (%)
Spouse	33 (72%)
Sibling	2 (4%)
Child	9 (20%)
Partner	1 (2%)
Niece	1 (2%)

**Table 2:** Demographic data (patients)

	Patient	
Sex	Number (%)	
female	24 (52%)	
male	22 (48%)	
Age (years)	Mean (min/max)	
	64 (35–88)	
		
Duration of care by the PCT (distribution)	Number (%)	
	less than 3 months:	24 (52%)
	3–6 months:	11 (24%)
	7–12 months:	5 (11%)
	more than 12 months:	6 (13%)
Duration of care by the palliative care team (weeks)	Median (min/max)	
	14 (4–250)	
Time from first diagnosis to death (years)	Median (min/max)	
	3.5 (0.5–16)	
Place of death	Number (%)	
At home	32 (70%)	
Palliative care ward	9 (20%)	
Hospital ward	1 (2%)	
Hospice	3 (6%)	
Nursing home	1 (2%)	

In the present study, the primary diagnosis was defined as the primary cancer disease (Table 3). Forty-one of the forty-six patients had at least one metastasis (89%); thirty-five patients had multiple metastases (85%).

**Table 3:** Primary diagnosis

Type of malignancy	Number (%)
brain tumor	5 (11%)
oropharynx tumor	5 (11%)
bronchial tumor	5 (11%)
breast cancer	4 (8%)
gastrointestinal tumor	11 (24%)
colorectal tumor	5 (11%)
urogenital tumor	7 (16%)
skin tumor	1 (2%)
malignancy of the hematopoetic system	3 (6%)

Forty-one patients (89%) were predominantly cared for at home for the entire course of the disease. Thirty-two patients (70%) were able to die at home (Table 2).

The patients had been attended by our palliative care team for an average of 26 weeks (range 4 to 250 weeks, median 14 weeks).

Emergency medical service was called 23 times in the period under investigation. Care-giving relatives of eighteen patients (39%) called the emergency medical service during the last six months of their lives for acute situations in their homes. Relatives of fourteen patients placed one call, three placed two calls and one placed three calls each during this period.

Of the total of 23 emergency calls registered during the study period, twenty-one (91%) were placed before the patients had started receiving support from the palliative care team. Two of the emergency calls (9%) registered in the present study were placed for patients already tended to by our palliative care team. One patient with diagnosed bronchial cancer was experiencing acute dyspnea. At the time of the call, his primary caregiver (his wife) was not present at the time and was thus unable to implement the defined emergency plan. The substitute caregiver was overwhelmed by the situation and placed the emergency call. The emergency call was placed for the second patient when he became unconscious. The palliative care team was informed simultaneously and arrived promptly but too late to cancel the emergency call. Death was determined by the emergency physician in the presence of the members of our palliative care team. Supportive care was given to the surviving relatives by a nurse of our palliative care team.

In sixteen of the 23 emergency calls (70%), the patient was admitted to the hospital; however neither of the two emergency calls for patients already in palliative care (one for severe dyspnea, one for unconsciousness for unknown reason) led to hospital admittance.

Acute dyspnea was the most common reason for contacting the emergency medical service (39%) followed by acute exacerbation of pain in six cases (26%; Table 4). All emergency calls were related to an acute situation directly or indirectly caused by the underlying cancer disease.

**Table 4:** Main reason for emergency call

Symptom	Number (%)
Respiratory distress	9 (40%)
pain	6 (26%)
seizures	4 (17%)
unconsciousness	3 (13%)
fracture	1 (4%)

All patients were in home care at the time of the emergency call. For seven patients, the emergency call not only led to hospital admission with a change in primary caregivers, but also to the patient not being able to die at home. Five of these patients died in a palliative care unit, one patient in a general hospital ward and one patient died in a hospice.

Twenty-nine of the interviewed relatives (63%) were unable to imagine home care and nursing of the patient in the terminal phase without the support of our palliative care team. The most important reason given for this opinion was that someone was always available to support them. In addition, thirty interviewees (65%) expected from the palliative care team patient-adapted, adequate symptom control and support in caring for the patient at home.

The average MCS-12 score (subscore of SF-12^®^) of the interviewed relatives was 41 (range 28–57, median 39) indicating that the state of psychological strain on the caregivers was significantly higher (p = 0.0043) than in the general population (median 52, range 46–60; [16]). The MCS-12 scores of the caregiving relatives who had called the emergency services during the last six months of the patients' lives did not indicate a higher level of psychological strain than in the relatives who had not placed an emergency call (median 37 vs. 43; p > 0.05).

The subjective assessment of the psychological stress (evaluation by self constructed questionnaire) present at the time of the emergency call showed a positive correlation with the MCS-12 scores. There was no statistically significant difference between the subjective assessment and the objective MCS-12 score (p > 0.05).

### Correlations

No significant correlation could be found between the number of emergency calls and how long the patients had been attended by their relatives and our palliative care team (p > 0.05). There was no correlation concerning the MCS-12 Score and the psychosocial stress factor of the caregiving relatives

However, support by our palliative care team significantly reduced the number of emergency calls during the patients' final six months of life (p = 0.032).

## Discussion

The present study shows the influence of our palliative care team during the last six months of life with regard to reducing the number of emergency calls by palliative cancer patients and their relatives. Therefore, the study approaches the important issue of whether and how our palliative care team could prevent unnecessary hospital admissions towards the end of life.

Approximately 3–4% of all emergency calls are for palliative patients in the final stages of their disease [[8,17], and [18]]. Most of these calls were placed while the patients and their care-giving relatives had no access to a palliative care team [17]. The present survey showed that most emergency calls in the last six months of the terminally ill patients' lives were placed before the patient was in the care of our palliative care team (91% vs. 9%). Thirty-five of the patients had been in the care of the palliative care team for less than six months. None of the eleven patients who had been seen by the palliative care team for more than six months placed an emergency call. This finding suggests that emergency medical services tend to be called in less frequently if the patient and the caregiving relatives can have the continuous and always available support by competent palliative caregivers. In the present investigation, only two (9%) of the emergency calls were placed while the patients had access to the palliative care team.

Acute situations can arise in the end-of-life phase of palliative patients that require a prompt reaction. In the final stage of cancer disease, symptoms can exacerbate or new, acute problems can arise that emphasize the dynamics of the disease process [19]. Caregiving relatives can be under great physical and psychological strain in such an acute situation. Therefore, this psychosocial strain can be overwhelming and can induce the caregiver to call the emergency medical service when what they actually want is advice and someone to talk to in a situation they experience as threatening and confusing. One should not ignore the fact that life-threatening situations can occur (e.g. acute hemorrhage, suffocation) that do require immediate medical attention. However, symptoms usually worsen gradually, giving time to make contingency plans. Due to their state of overwhelming psychological strain the caregivers are often unable implement and follow these plans. On the other hand, some symptoms cannot be dealt with at home under the best of circumstances.

The following structural approaches for improving the emergency medical care of palliative care outpatients are under discussion:

Early studies also show that most emergency calls are made by or for patients, for whom outpatient palliative care is not available [8,10]. The results of the present study support these findings. The merit of continuous support during the end-of-life with regard to the frequency of emergency calls has also been reported by other investigators [6]. When these services are unavailable, the caregiving family members are left on their own, and this often leads to overstrain and a lack of cooperation. Emergency physicians on the scene have to decide on the spot without knowledge of the patient's wishes and existing contingency plans or therapy restrictions. For this reason it is essential to integrate palliative medical topics into the curriculum for emergency medicine to ensure patient-oriented therapy [[8,9,17,20,21] and [22]]. In Germany, this might mean including topics from the specialty training guidelines for palliative medicine described in the regulations for further training set forth by the German Medical Association in the requirements for the specialty of emergency medicine [9].

On the other hand, it is eminently important to recruit professional palliative medical assistance at an early stage of the disease, as this can positively influence the fulfillment of the patient's wishes in the final stage of their disease [10,11].

A further possibility for improving the emergency care of palliative outpatients is to integrate a palliative care team into the primary alert structure of the emergency medical service at the level of the dispatcher [11,23]. The idea of this would be that a palliative care team would be alerted at the time of an emergency call from caregivers of palliative patients as part of the primary response. The palliative care team could then visit the patient at his home. The key word for the dispatcher might be "cancer patient", "palliative care patient" or "hospice patient". A second alternative would be for the emergency medical service primarily seeing the patient to alert the palliative care team who would then take over the outpatient care. A study of this model in France showed that there was a significant reduction in the number of hospital admissions [11,23].

Measures should be initiated within the ambulatory care organizations aimed at eliminating the information gaps often encountered in the care of patients with life-threatening disease. In this context, it has been suggested that emergency plans be prepared [8,17] or that a copy of the patient's medical file be left with the patient [24] in order to provide the emergency physician treating the patient in the acute situation with information on the disease, its course and therapy decisions. Closing information gaps is considered to be pivotal in improving medical care at the end of life, since inappropriate decisions at this point are often the result of information deficits [22,26].

Apart from the influence of pertinent information on therapy decisions in emergency situations, another successful measure has been to supply the patient with an emergency medicine chest containing medications that he might require in an acute situation. The caregiving relatives are trained to administer the medications in precisely defined situations in order to act according to the patient's wishes and alleviate symptoms and suffering in the acute situation [24]. One could object to this practice on the grounds that it is exactly in such extraordinary situations that the relatives do not follow or are unable to follow the therapy plans laid out and agreed upon beforehand. Further studies are necessary to determine which factors determine success and failure.

A further way to ensure that medical care follows the will and wishes of the patient is to draw up an advance directive, give instructions for critical situations, such as a "Do not attempt resuscitation" (DNAR) order, and to grant power of attorney. This can assist the emergency physician in his treatment decisions [[8,23,27], and [28]]. Correctly worded and presented at the proper moment, these can have a decisive influence on the patient's further treatment [29]. Outpatient care by a palliative care team would not only reduce the frequency of emergency calls by patients on palliative care, but it would also have the effect that patients would not have to be admitted to the hospital and could remain in their homes to die as they wished [30].

A secondary effect of these structural approaches is that a therapy concept adapted to the wishes of the patient does not have to be abandoned due to emotional exhaustion of the caregiver, since the latter has been shown to be one of the primary reasons for contacting the emergency medical service [[8,31], and [32]]. This kind of stressful situation, which can overstrain the caregiver and thereby trigger irrational decisions, will be intensified by a lack of adequate outpatient support [[11,23,31], and [32]]. A palliative care team like the one participating in this study, which is available 24 hours every day, can contribute to keeping the patient in his domestic environment for therapy and care even in critical situations. By continuously supporting the caregivers and preparing them for acute events, one can often reduce the impact of and put into perspective the situations that might otherwise have led them to make an emergency call. This cannot always prevent palliative medical emergencies, but their effects on patient-oriented care can be attenuated. This is reflected by the assessment of 29 of the interviewed family members (63%), who stated that care of the patient would not have been possible at home without the support of our palliative care team.

The acute situations described in the present study placed an overwhelming strain on the relatives, which led them to call the emergency medical service. Critical situations also arose during the time the patients were being cared for by our palliative care team, but the psychological burden of the caregiving relatives was markedly reduced. The MCS-12 scores were significantly lower amongst the caregiving relatives of cancer patients indicating greater mental strain compared to the general population [16]. A comparison with published data showed that family members who had the support of our palliative care team tended to be under less mental strain than those who cared for tumor patients without support of the palliative care team [16]. However, the present study did not show any connection between low MCS-12 scores of the caregivers and the likelihood of their placing an emergency call. The relevance of the measured MCS-12 scores for predicting the likelihood of an emergency call is questionable, since they were determined retrospectively some considerable time after the emergency situation. One might conclude from the significantly lower frequency of emergency calls after institution of support by the palliative care team that this support had also led to a reduction of the psychological burden on the caregivers. The fact that critical situations and with them reasons for calling the emergency medical service occur more often during the final stages of the disease [19,33] lends additional weight to this observation.

The reasons for calling the emergency medical service found in the present survey were the same frequently given in other studies of palliative care patients [8,19,34-37]. Therefore, the findings of this investigation seem to be generalisable.

## Limitations of the study

In the present investigation we included only cancer patients. At the time of our study only palliative patients with cancer disease and not patients with other life limiting illnesses (e.g. end-stage heart failure, end-stage neurological failure) were treated by our palliative care team.

All patients had cancer diseases. During the defined period our palliative care team treated only cancer patients. Therefore, other concomitant conditions (e.g. pre-existing neurological or cardiac disease) were considered irrelevant for the purpose of the study and were not taken into consideration.

The retrospective design of our investigation may not show all reasons for calling the emergency medical service during an acute situation.

The SF-12^® ^was created as subscore of the SF-36^® ^to evaluate mental and physical strain of caregiving relatives. The evaluation of these items should be prospective. In our study we evaluated the MCS-12 retrospectively. A retrospective analysis of the MCS-12 is not as valid as a prospective analysis. Therefore, the mental strain might be still substantially more distinctive at the caregiving time.

We have to mention that there are differences in emergency medical services all over Europe. In Germany there is a limited paramedic system and therefore the emergency physician goes to the patient by an ambulance; this fact is not the case in all countries.

## Conclusion

The present study showed that emergency medical services were called significantly less frequently in acute situations when our palliative care team was involved in the patient's medical care. This allows for patient-oriented care of life-threatening chronic diseases, even in the final stages.

Calls for emergency medical care of palliative patients in the final stage of their disease are not uncommon in Germany and occur at a rate similar to that for emergencies in infants. Therefore, changes and improvements in the structure of caregiving can reduce the number of emergency calls for these patients and can help avoid unnecessary hospital admissions. However, training and continuous medical education of emergency physicians and paramedics, the communication and cooperation between palliative care teams and the emergency medical services with regard to end-of-life therapy concepts are necessary. It is also necessary to integrate palliative care teams into the primary emergency care of palliative patients as soon as possible. An emergency plan and emergency medications for the caregiving relatives which are prepared for possible emergency situations may be drawn up. Last but not least an advance directive should be drawn up by the patient.

Therefore, hospital admissions, which are often not desired by cancer patients in the final stage of their disease, can be reduced considerably.

## Competing interests

All authors declare that they have no competing interests. The manuscript contains parts of a master's thesis (A. Vossen-Wellmann; Universitaire Kurt Bösch Sion) and some of the data were previously presented at the congress of the European Association for Palliative Care in Budapest 2007.

## Authors' contributions

CHRW and GGH participated in designing the study. CHRW, AV–W, and HCM participated in collecting and entering the data. AFP supported in editing the manuscript. BMG co-wrote the manuscript and added important comments to the paper. All authors read and approved the final manuscript.

## Pre-publication history

The pre-publication history for this paper can be accessed here:


